# Identification of candidate genes associated with resistance against race 0 of *Colletotrichum lentis* in *Lens ervoides*

**DOI:** 10.1038/s41598-022-23175-z

**Published:** 2022-11-02

**Authors:** P. K. Bawa, J. Halliday, K. Kapoor, S. Banniza

**Affiliations:** Crop Development Centre, Department of Plant Sciences, University of Saskatchewan, Saskatoon, SK Canada

**Keywords:** Plant breeding, Plant molecular biology, Plant stress responses

## Abstract

Resistance to anthracnose caused by the fungal pathogen *Colletotrichum lentis* was explored through transcriptome sequencing over a period of 24 to 96 h post-inoculation (hpi) of the partially resistant recombinant inbred lines (RIL) LR-66-528 and susceptible LR-66-524 of the crop wild relative *Lens ervoides* population LR-66. The development of infection vesicles and primary hyphae by *C. lentis* were significantly higher on susceptible RIL LR-66-524 compared to partially resistant LR-66-528 at 24 and 48 hpi, but exponential trends in fungal growth were observed between 24 to 96 hpi in both RILs. Comparison of inoculated with mock-inoculated samples revealed 3091 disease responsive genes, among which 477 were differentially expressed between the two RILs. These were clustered into six expression clusters with genes that had either high or low expression in one of the RILs. Differentially expressed genes (DEGs) were functionally annotated and included genes coding LRR and NB-ARC domain disease resistance proteins, protein detoxification, LRR receptor-like kinase family proteins, and wall-associated Ser/Thr Kinases. DEGs were compared to genes in previously published anthracnose resistance QTLs mapped in LR-66 and revealed 22 DEGs located in 3 QTLs. Expression of 21 DEGs was validated using RT-qPCR confirming expression trends in RNA-seq.

## Introduction

Anthracnose caused by the hemibiotrophic fungal pathogen *Colletotrichum lentis* Damm is currently the most important foliar disease of cultivated lentil (*Lens culinaris* Medik.) in Canada, where it was first discovered in the province of Manitoba in 1987 before spreading into Saskatchewan in the 1990s^1^. It has also been reported from lentil crops in the USA, Bulgaria, Pakistan and New Zealand^2^. Yields losses of up to 100% have been encountered in worst case scenarios triggered by short crop rotations, frequent rainfall and high temperature^3^. Two pathogenic races of *C. lentis* infect the lentil crop in Canada^4^. Race 0 is dominating and more aggressive, and no high levels of resistance have been identified in cultivated lentil, whereas partial resistance to race 1 was identified and incorporated in lentil cultivars^5^. Wild relatives of crop species are repositories of allelic diversity for disease resistance, and accessions of *Lens ervoides* (Brign.) in the tertiary gene pool of *Lens* with superior resistance to anthracnose were discovered^6^. Using interspecific hybridization involving embryo rescue, transfer of resistance to *C. lentis* race 1 and race 0 from *L. ervoides* accession L01-827A to lentil cultivar Eston was achieved^7,8^. However, lack of understanding of the mechanisms and control of resistance has prevented the development of markers to trace the introgression of genes into breeding lines. The intraspecific recombinant inbred line (RIL) population LR-66 was developed from crossing *L. ervoides* accessions L01-827A and IG 72,815^9^ and allowed for the identification of QTLs conferring resistance to anthracnose through phenotyping and SNP-based genotyping^10^. In this study, the development of *C. lentis* on and in leaflets was investigated for two RILs of LR-66 with contrasting levels of resistance to identify specific stages of fungal growth for a transcriptome study. An RNA-seq time-series experiment was conducted on these two *C. lentis*-infected RILs to identify differentially expressed genes (DEGs) to gain an overview of host responses during infection.

## Results

### Quantification of fungal development

**Figure 1:**
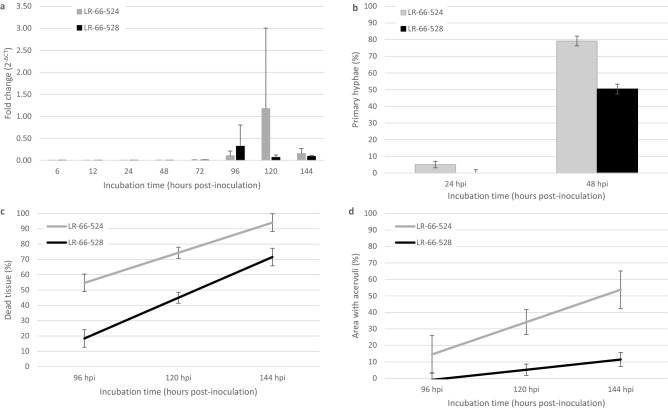
Quantitative parameters assessed for the infection process of *Colletotrichum lentis* race 0 isolate CT-30. Infection structures and symptoms were assessed in susceptible recombinant inbred lines LR-66-524 and partially resistant LR-66-528 of *Lens ervoides* population LR-66*.* (**a**) Relative fungal biomass of *C. lentis* expressed as the Log2 fold change of *C. lentis* *ClACT* (determined by qPCR) relative to mock (non- inoculated) samples. Error bars represent standard deviations of three biological replicates. (**b**) Proportion of infection vesicle (IV)/primary hyphae (PH) per 25 appressoria from 24 to 48 hpi. (**c**) Percentage of dead tissue per leaflet from 96 to 144 hpi. (**d**) Percentage of leaflet area covered with acervuli. (**b–d**) Error bars represent standard errors of the means of 3 biological replicates.

The development of *C. lentis* isolate CT-30 was studied in susceptible *L. ervoides* RIL LR-66-524 and partially resistant LR-66-528 through histopathological observations and an assessment of fungal biomass, estimated through quantification of genomic *C. lentis ACTIN* relative to the *L. culinaris* housekeeping gene Elongation factor 1-alpha (*EF1-α*) by qPCR. Appressoria formation was observed by 6 hpi in both genotypes but at variable numbers. Fungal biomass did not proliferated much from 6 to 72 hpi (Fig. 1a), most of which represented the biotrophic phase of *C. lentis*. Infection vesicle/primary hyphae were visible beneath some appressoria in susceptible LR-66-524 by 24 hpi, but not in partially resistant LR-66-528, where they had formed by 48 hpi (Fig. 1b). More infection vesicle/primary hyphae were observed in the susceptible than in the partially resistant RIL. A dramatic increase in fungal biomass was observed at 120 hpi in the susceptible RIL LR-66-524, whereas it peaked at a lower level at 96 hpi in the partially resistant RIL LR-66-528 (Fig. 1a). However, significant differences were only observed at 24 hpi. Secondary hyphae were not visible as they did not stain well during sample preparation. The percentage of dead tissue and the percentage of leaflet area covered by acervuli per leaflet due to infection increased with incubation time (Fig. 1c,d). There was significantly more dead leaf tissue in susceptible LR-66-524 than in partially resistant RIL LR-66-528 at 96, 120 and 144 hpi, and more leaflet area covered by acervuli at 120 and 144 hpi. Although fungal growth appeared to level off after 96 h in both RILs, there was a trend for higher biomass of *C. lentis* in susceptible RIL LR-66-524 as compared to partially resistant RIL LR-66-528 (Fig. 1a). Based on these observations, 24, 48, 72 and 96 hpi were selected for gene expression studies.

### Analyses of transcriptome variability between RILs

**Figure 2:**
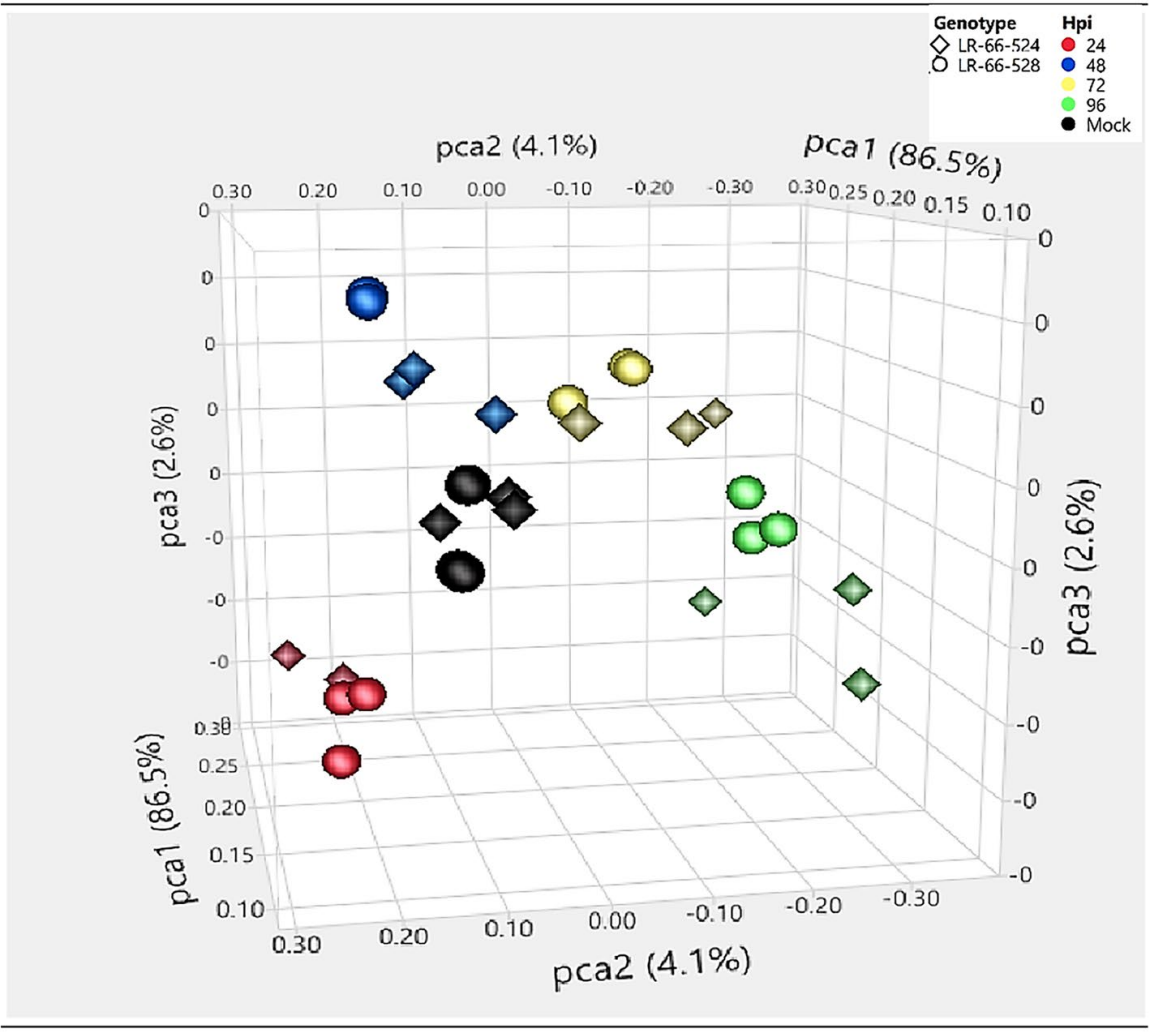
Three-dimensional Principal Component Analysis plot representing the variability among sequencing samples. RNA-seq data were generated from partially resistant recombinant inbred lines RIL LR-66-528 and susceptible RIL LR-66-524 of *Lens ervoides* population LR-66 inoculated with race 0 isolate CT-30 of *Colletotrichum lentis* and incubated for 24, 48, 72 and 96 h. Data is represented in x-y-z coordinates with principal component axes (pca1, pca2 and pca3) representing genotypes, biological replicates and time-points.

RNA sequencing from inoculated leaves of susceptible RIL LR-66-524 and partially resistant RIL LR-66-528 sampled 24, 48, 72 and 96 hpi generated approximately 10–14 million high-quality raw reads as paired-end sequences in a FASTQ format with a length of 125 bp per library. The STAR aligner output showed that approximately 80–85% of the filtered reads aligned to the annotated *L. culinaris* genome. Most of the remaining reads were assumed to be of fungal origin, and some were probably specific to *L. ervoides*. The read counts file generated during the mapping process had more than 30,000 genes. The gene read counts were used to analyze the transcriptome variability among the samples. Principal Component Analysis revealed increasing differences between the RILs as time after inoculation increased (Fig. 2). Principal Variance Component Analysis conducted on samples of the two RILs collected in replicates at 24, 48, 72 and 96 hpi indicated that they collectively accounted for 79.9% of the total variance. The largest variance proportion (69.8%) could be attributed to incubation time (hpi), followed by the hpi x RILs (1.8%) and block (0.8%), whereas RILs only contributed 0.4%.

**Figure 3:**
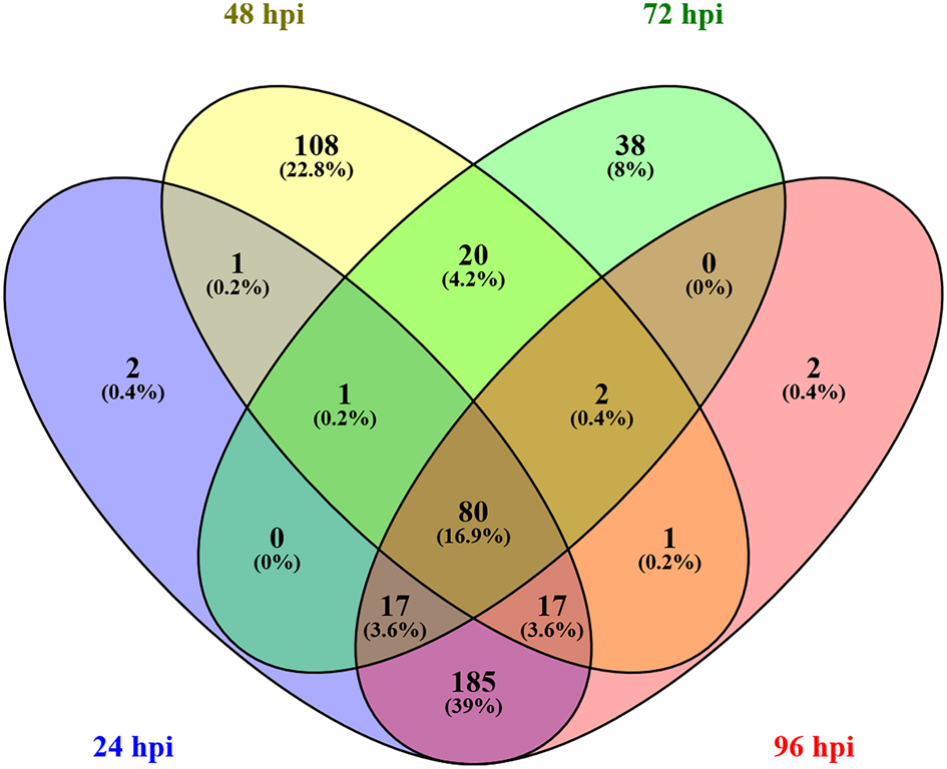
Venn diagram showing the number of genes differentially expressed between partially resistant recombinant inbred line LR-66-528 and susceptible RIL LR-66-524 of *Lens ervoides* population LR-66 in response to inoculation with race 0 isolate CT-30 of *Colletotrichum lentis* and incubated for 24, 48, 72 and 96 h. Violet color represents genes differentially expressed between LR-66-528 and RIL LR-66-524 exclusively at 24 hpi, yellow for genes at 48 hpi, green for genes at 72 hpi and pink for genes at 96 hpi. Genes in overlapping areas are differentially expressed between LR-66-528 and RIL LR-66-524 at more than one time-point.

Disease responsive DEGs between the two RILs were separated from other differentially expressed genes by comparing inoculated with mock-inoculated samples. As a result, 3091 genes were identified with a fold change larger than 2 (Padj < 0.05) compared to mock-inoculated samples (Supplementary Table S1). Interestingly, in the partially resistant RIL LR-66-528, 2511 disease-responsive genes were expressed each at 24 and 48 hpi compared to 352 and 561 in the susceptible LR-66-524, whereas that trend was reversed by 96 hpi when 2511 disease-response genes were expressed in LR-66-524 compared 1968 in LR-66-528. Among all of the 3091 disease responsive DEGs, 477 genes were differentially expressed between the two RILs (fold change > 2, Padj < 0.05, Supplementary Table S2) indicating that 85% of disease-responsive genes were commonly expressed by both RILs. Among the 477 genes, 80 genes were differentially expressed between the two RILs during the entire period lasting from 24 to 96 hpi (Fig. 3). Only two genes were differentially expressed between the two RILs exclusively at 24 hpi or at 96 hpi, whereas 108 were at 48 hpi and 38 genes at 72 hpi. Up to 185 genes were differentially expressed between the two RILs at more than one time point.

**Figure 4:**
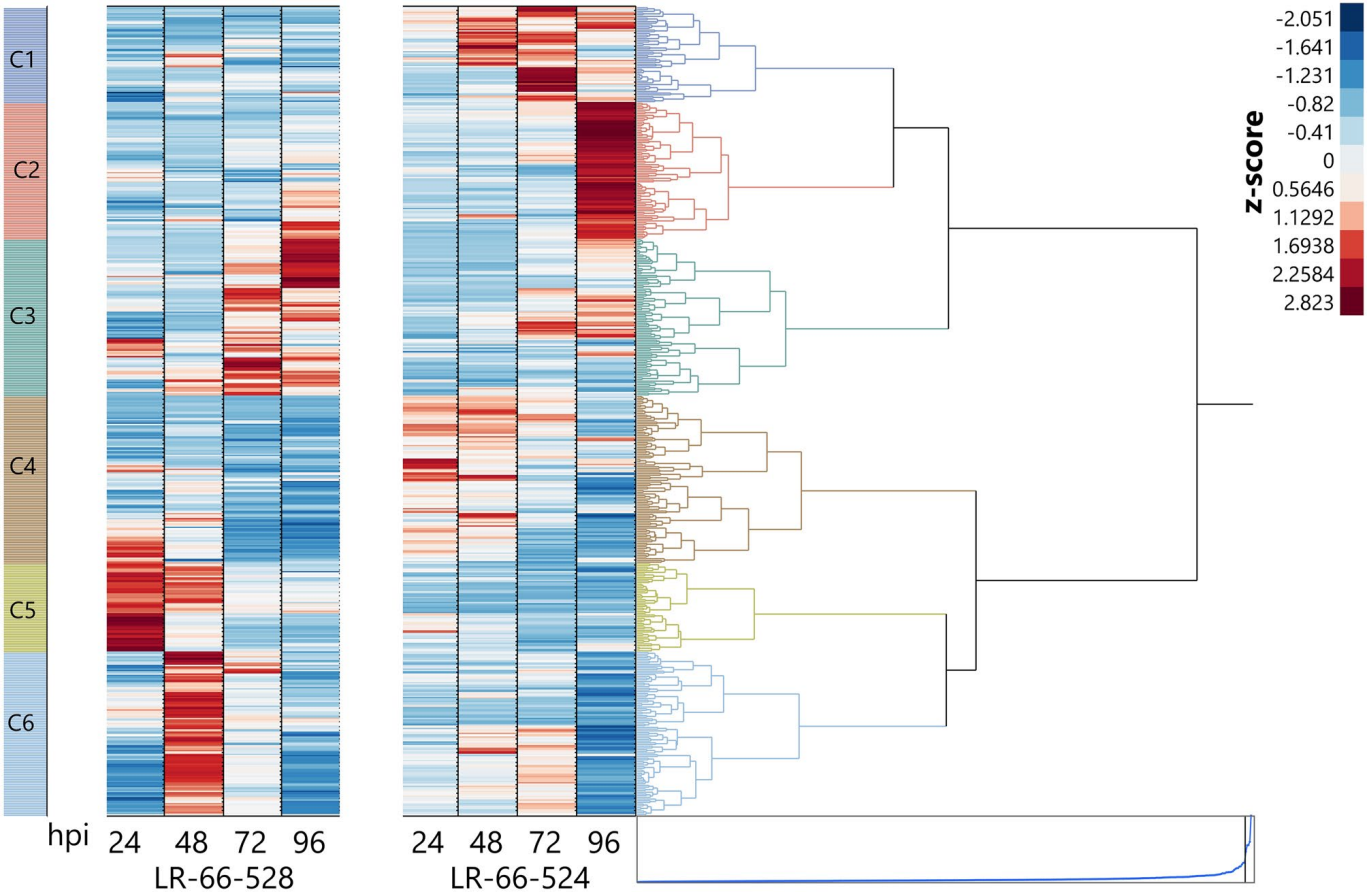
Hierarchical cluster analysis of gene expression profiles of differentially expressed genes. Genes were differentially expressed between partially resistant recombinant inbred line LR-66-528 and susceptible RIL LR-66-524 of *Lens ervoides* population LR-66 in response to inoculation with race 0 isolate CT-30 of *Colletotrichum lentis* and incubated for 24, 48, 72 and 96 h. C1 to C6 represent different clusters based on the expression of genes. Heat map shows the normalized expression levels of transcripts represented by a color spectrum ranging from red (high expression levels) to blue (low expression levels). The dendrogram shows Pearson’s correlation with an average distance among clusters.

The 477 DEGs clustered into six expression clusters based on K-mean clustering (Fig. 4). Cluster 1 contained 56 genes, 75% of which had high expression as indicated by z-scores higher than zero at 48, 72 and 96 hpi in susceptible RIL LR-66-524, compared to 21% in partially resistant LR-66-528. All of the 80 genes in Cluster 2 had z-scores higher than zero at 96 hpi in LR-66-524, whereas that ranged from 12 to 37 at the other time points for this genotype, and from six to 50 in LR-66-528. Among a total of 92 genes in Cluster 3, 86 and 87 had z-scores higher than zero in the partially resistant RIL LR-66-528 primarily at 72 and 96 hpi, respectively, compared to a high of 60 genes in LR-66-524 at 96 hpi. Cluster 4 contained 98 genes that were highly expressed in LR-66-524 primarily at 24 (90 genes with z-scores > 0) and 48 hpi (83 genes with z-scores > 0), compared to 34 genes with z-scores higher than zero in LR-66-528 at those time points. Cluster 5 was the smallest of all the clusters consisting of 52 genes, all of which had z-scores higher than zero in LR-66-528 at 24 hpi, and 45 genes at 48 hpi, compared to 17 and 2 genes for those two time points in LR-36 66-524. Cluster 6 included 96 genes highly expressed in the partially resistant RIL LR-66-528 with *z* scores higher than zero for all genes at 48 hpi and 64 genes at 72 hpi, whereas in LR-66-524 this was only the case for 43 at 48 hpi and 59 genes at 72 hpi.

The results of gene ontology showed that a total of 370 out of 477 DEGs had homology with the *M. truncatula* genome (Supplementary Table S2), among which 159 could be assigned to GO terms in different hierarchical clusters and were involved in different biological processes (Fig. 4, Supplementary Table S3). The GO terms for genes in Cluster 1 showed that these were enriched in cell death related processes such as “programmed cell death”, “regulation of cell death”, and in cell wall organization or biogenesis such as “cell wall macromolecule metabolic process”. Enriched GO terms for Cluster 2 were involved in responses to stress such as “response to water”, “response to water deprivation”, and cell morphogenesis involved in differentiation such as “pollen tube growth” and “cell tip growth”. GO terms for genes in Cluster 3 were enriched in protein phosphorylation processes such as “peptidyl-tyrosine dephosphorylation” and RNA processing such as “mRNA splicing, via spliceosome”, RNA splicing, via transesterification reactions” and “RNA splicing, via transesterification reactions with bulged adenosine as nucleophile”. GO mapping of genes in Cluster 4 showed that these were enriched for genes with function in cell growth and differentiation processes such as “cellular developmental process”, cellular biogenesis such as “plant-type secondary cell wall biogenesis”, “regulation of cellular component biogenesis”, and metabolic processes such as “hormone metabolic process” and “regulation of hormone levels”. GO terms for genes in Cluster 5 were enriched in carbohydrate and amino acid metabolic processes such as “oligosaccharide biosynthetic process” and “aspartate family amino acid metabolic process”. Go terms for genes in Cluster 6 were enriched with stress-related process such as “response to water”, DNA metabolic process such as “DNA recombination”, regulation of hydrolase activity such as “regulation of GTPase activity”, DNA packaging processes such as “chromatin assembly” and “nucleosome organization”.

### Comparison of differentially expressed genes with QTLs

The projection of anthracnose resistance QTLs *qANTH0-2*, *qANTH1-2.1* and *qANTH1-2.2* present on linkage group 2^10^ of the *L. culinaris* linkage map onto the physical map led to the identification of 3078 genes. The QTL intervals *qANTH0-3*, *qANTH1-3.1* and* qANTH1-3.2* on linkage group 3 contained 605 genes, and those of *qANTH0-5.1*, *qANTH0-5.2*, and *qANTH1-5.2* present on linkage group 5 had 1437 genes. The projection of *qANTH0-7* localized on linkage group 7 had a total of 713 genes. Comparison of these genes with DEGs identified by RNA-seq led to the identification of 22 DEGs, of which 9 DEGs were found on chromosome 2, 10 DEGs on chromosome 5 and 3 DEGs on chromosome 7 (Table 1). Two of these genes, *Lc23518* and *Lc09295*, were included in the gene expression validation through RT-qPCR. Based on RNAseq analysis, *Lc23518* had low expression at 24 hpi and was highly expressed at 96 hpi in the partially resistant RIL and was found in QTL interval qANTH0-5.1 localized on linkage group 5. *Lc09295* was highly expressed at 96 hpi in the partially resistant RIL and was located in the QTL interval qANTH0-2 on linkage group 2.

**Table 1:** *Colletotrichum lentis-*responsive *Lens culinaris* genes differentially expressed between *Lens ervoides* recombinant inbred lines LR-66–524 (susceptible) and LR-66–528 (partially resistant) based on RNA-seq analysis, time post-inoculation (hpi), presence in resistance QTLs identified for *Lens ervoides* population LR-66^10^, their annotation, fold change and standard error (ifcSE) and adjusted P-value (padj).

Gene ID	hpi	QTL	Gene annotation	log2 fold change	lfcSE	padj
Lc09011	96	qANTH0-2	Uncharacterized protein	− 2.43	0.5970	0.0012
Lc09511	24	qANTH0-2	DEAD-box ATP-dependent RNA helicase, putative	2.05	0.6597	0.0215
	96					
Lc09494	24	qANTH0-2	C-repeat binding factor 3	− 1.25	0.6179	0.1802
Lc05315	24	qANTH0-2	DEAD-box ATP-dependent RNA helicase 27	− 0.73	0.6948	0.5403
	96					
Lc09713	24	qANTH0-2	Acetyl-CoA acetyltransferase, mitochondrial, putative	− 1.99	0.4235	0.0001
	96					
Lc05920	48	qANTH0-2	Lissencephaly type-1-like homology motif WD40-like	3.33	0.8883	0.0049
Lc06016	24	qANTH0-2	RNA 2’-phosphotransferase, Tpt1/KptA family protein	− 1.15	0.3615	0.0184
	96					
Lc09295	24	qANTH0-2	MYB transcription factor MYB91	1.61	0.5653	0.0405
	96					
Lc10375	24	qANTH0-2	Uncharacterized protein	1.91	0.4157	0.0002
	48					
	96					
Lc20642	24	qANTH0-5.1/5.2	Putative ribonuclease H protein	− 1.66	0.5215	0.0184
	96					
Lc23822	24	qANTH0-5.1/5.2	AFG1-family ATPase	1.93	0.4341	0.0003
	48					
	96					
Lc22530	24	qANTH0-5.1/5.2	PolI-like B DNA polymerase	− 6.49	1.3230	0.0000
	48					
	96					
Lc22194	24	qANTH0-5.1/5.2	Uncharacterized protein	− 1.40	0.4041	0.0087
	48					
	96					
Lc22494	24	qANTH0-5.1/5.2	Uncharacterized protein	− 1.10	0.3338	0.0142

### Validation of gene expression using RT-qPCR

**Figure 5:**
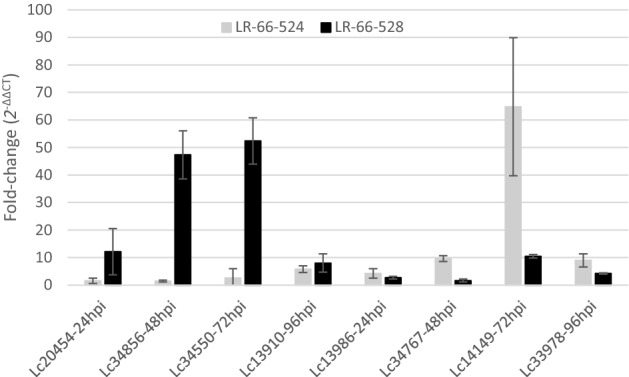
Fold changes in gene expression of selected genes and time points (hpi) after inoculation with *Colletotrichum lentis* in partially resistant *Lens ervoides* LR-66-528 and susceptible RIL LR-66-524 compared to mock-inoculated controls based on RT-qPCR. Fold changes (2^-ΔΔCT^) were calculated as described by Schmittgen and Livak^40^, using *EF1-α* for normalization. Bars indicate standard deviations. Lc20454: LRR receptor-like kinase, LC34856: F-box/LRR protein, Lc34550: LRR and NB-ARC domain disease resistance protein, Lc13986: Adenosylhomocysteinase (AdoHcyase) (3.3.1.1) (S-adenosyl-L-homocysteine hydrolase), Lc34767: TMV resistance protein N, Lc14149: LRR and ubiquitin-like domain plant-like protein, Lc33978: LRR receptor-like kinase.

Based on functional annotations indicating a putative role in disease responses, 21 DEGs were selected for RT-qPCR at one, two or three time points, for a total of 28 RT-qPCR reactions. Expression trends estimated through RT-qPCR were the same as those estimated through RNA-seq; however, for nine out of the 28 RT-qPCR tests, or 32%, differences in gene expression were not significant (Supplementary Table S4). Fold changes for the expression of eight DEGs, assessed through RT-qPCR, in the partially resistant LR-66-528 and the susceptible LR-66-524 compared to the mock-inoculated controls of both RILs are presented in Fig. 5.

## Discussion

The hemibiotroph *C. lentis*, causal agent of anthracnose, is the most important foliar pathogen of lentil in Canada due to limited resistance in the cultivated species. Resistance to the dominating and aggressive race 0 of *C. lentis* has been identified in *L. ervoides*^6^ and interest in introgressing useful genes from *L. ervoides* into elite cultivars has increased facilitated by embryo rescue techniques that overcome interspecific reproductive barriers between *L. ervoides* and *L. culinaris*^7^. Nevertheless, progress in developing partially resistant varieties has been slow, to a large part because an understanding of resistance mechanisms and their underlying genes are lacking. Previously, five QTLs were associated with anthracnose resistance on a SNP-based linkage map of the F_9_ recombinant *L. ervoides* inbred line population LR-66^10^. However, the direct use of these QTLs for marker-assisted selection has been limited and identification of candidate genes associated with resistance has been difficult because of the relatively large size of QTL intervals and linkage drag in interspecific progeny.

In the current study, anthracnose development and gene expression in responses to infection were further explored on the most resistant and most susceptible RILs of LR-66. The differentiation of appressoria, infection vesicles and primary hyphae are the first crucial steps in the invasion of the host plant by *C. lentis.* Histopathological examination revealed that infection vesicles and primary hyphae were still absent in the partially resistant LR-66-528 at 24 hpi, but had already differentiated in the susceptible LR-66-524, indicating that penetration of *C. lentis* isolate CT-30 may have been delayed or the success rate may have been much lower in the partially resistant LR-66-528 compared to the susceptible RIL. A similar observation was made in the *Arabidopsis thaliana*—*Colletotrichum higginsianum* host-pathogen system where more than 50% of appressoria initiated successful penetrations through the leaf surface to form primary hyphae in susceptible accession Ler-0, compared to 10% of appressoria on partially resistant accessions Ws-0, Gifu-2, Can-0 and Kondara^11^. As a hemibiotroph, this initial biotrophic phase is essential for pathogenesis, after which *C. lentis* switches to necrotrophic growth*,* visually evident here in the development of cell death starting at 96 hpi. Compared to the partially resistant RIL LR-66-528, cell death was significantly more pronounced in the susceptible LR-66-524 and more leaflet area was covered with acervuli, the fruiting structures of *C. lentis,* signaling that *C. lentis* was more successful at completing its life cycle on LR-66-524 than on LR-66-528. Differences in cell death, lesion number, lesion size and colonization efficiency of moderately virulent *C. lentis* isolate JPPTNL 882 were previously observed on susceptible *L. culinaris* cv. Eston compared to partially resistant lentil line PI 320,937 at 72 to 144 hpi, whereas there were no differences for highly virulent isolate 95S29, which was probably a race 0 isolate similar to CT-30 used in the current study^12^. Adding to histopathological observations, determination of fungal biomass indicated a dramatic increase after 48 hpi in both RILs, with a trend in the partially resistant LR-66-528 to accommodate less *C. lentis* than the susceptible RIL LR-66-524. It was previously observed that *C. lentis* switched from the biotrophic to the necrotrophic phase after 48 hpi^12,13^, so exponential growth of *C. lentis* based on qPCR would correlate with that switch from biotrophy to necrotrophy.

A total of 477 genes among 3091 disease-responsive genes (15%) were found to be differentially expressed upon pathogen infection between partially resistant LR-66-528 and susceptible LR-66-524. The large number of common genes in both RILs responding to infection by *C. lentis* are probably involved in basal defense reactions, whereas the genes differentially expressed between the two lines are associated with the different levels of resistance in the RILs, which was much higher in LR-66-528 (5% disease severity) compared to LR-66-524 (72%)^10^. Separation of sequencing samples in the PCA plot were evident at 48 and 96 hpi and indicated that transcriptome responses of LR-66-528 and LR-66-524 to *C. lentis* infection diverged after 24 hpi. Samples of both genotypes from 24 hpi were positioned far from those collected at 48, 72 and 96 hpi and correlated with the progression from biotrophy to necrotrophy. PVCA showed that the highest proportion of the variance (69.8%) could be attributed to incubation time. The percentage of the variance attributed to genotypes was the lowest (0.4%) among all principal components, indicating that difference in the host responses were subtle. A much higher proportion of the variance (15%) could be attributed to two other RILs of LR-66 responding to the necrotrophic pathogen *S. bortryosum*^14^.

Two DEGs, *Lc33118* and *Lc27093* were expressed exclusively at 24 hpi and were highly expressed in the partially resistant RIL LR-66-528. Functional annotations of these genes determined that *Lc33118* encodes a cytokinin receptor histidine kinase. Cytokinin and their receptors, histidine kinases, are involved in diverse developmental functions in plants, but also play a role in responses to pathogens where they were shown to positively and negatively regulate the development of biotrophs^15^. Treatment of *Arabidopsis* plants with exogenous cytokinin benzyl adenine (BA) 48 h prior to inoculation with the biotrophic pathogen *Hyaloperonospora arabidopsidis* at low concentrations increased susceptibility of the plants whereas high levels of BA increased resistance^16^. Cytokinins can also be secreted by certain pathogens to manipulate the host cell environment to its advantage thereby functioning as a virulence factor^17^. This was shown for *C. graminicola* and was associated with typical symptoms for high cytokinin referred to as green islands on senescing maize leaves^17,18^. A search of the *C. lentis* genome revealed that the enzyme isopentenyltransferases, which catalyses the first step in cytolkinin synthesis, is also present in this pathogen; however, high expression of the putative cytokinin receptor *Lc33118* at 24 hpi in the partially resistant LR-66-528 here indicates that an increase in endogenous rather than pathogen-derived cytokinin may contribute to resistance in this RIL. This was further supported by the simultaneous high expression levels of *Lc37093* in this RIL, which showed features of a CAP/cysteine rich secretory/antigen 5 protein. These proteins belong to the CAP protein superfamily (Cysteine-rich secretory proteins (CRISPs), Antigen 5 (Ag5), and Pathogenesis-related 1 (PR-1) proteins), proteins of which have been identified in animals, plants and fungi^19^. The most studied CAP proteins in plant defense are pathogenesis-related 1 (PR1) proteins^20^, and indeed, *Lc37093* had the highest similarity with MtSTS14 (a PR-1 protein) in *Medicago*. In *Arabidopsis*, it was demonstrated that cytokinin and SA together strongly activate PR-1 expression through the transcription factor TGA3^21^.

The highest number of DEGs (108) exclusively expressed at any one time point were identified at 48 hpi, which coincides with the biotrophy-necrotrophy switch. Many of these genes were highly expressed in the partially resistant RIL LR-66-528 and were associated with stress-related processes, DNA metabolic processes, the regulation of hydrolase activity and DNA packaging processes. Among these DEGs were also genes that regulated or coordinated the SA and ET/JA pathways, both of which are typically engaged in response to hemibiotrophs. Among the 38 DEGs exclusively expressed at 72 hpi, *Lc29695*, a disease resistance protein (TIR-NBS-LRR), and *Lc33733*, a Pentatricopeptide-repeat proteins (PPRs) containing plant protein, were highly expressed in the partially resistant RIL LR-66-528. PPRs were previously shown to be upregulated in rice in response to inoculation with *Magnaporthe oryzae*^22^. At 96 hpi, among numerous disease-responsive LRR receptor-like kinase family proteins, one (*Lc34740*) was expressed at high level at this time point in LR-66-528. Among many other roles, some LRR receptor-like kinase family proteins function as pattern recognition receptors (PRRs) that recognize pathogen associated molecular patterns (PAMPs) thereby regulating plant immune responses against invasive microorganisms^23^.

Other genes primarily highly expressed in the partially resistant RIL LR-66-528 were associated with RNA splicing (Cluster 3), the primary metabolic and transportation processes (Cluster 5) and processes such as “regulation of GTPase activity” (Cluster 6). While the up-regulation of the primary metabolism to modulate signal transduction cascades and enhances plant defense responses has been well studied^24^, the role of RNA splicing, in particular of long non-coding RNAs, in plant defense has only emerged more recently^25^. In a recent differential transcriptome study of walnut infected with the hemibiotroph *C. gloeosporioides*, approximately 5% of transcripts were non-coding and almost 24% were long non-coding RNAs, many of which were differentially expressed between a partially resistant and susceptible genotype at various time points^26^. Much better understood is the role of GTPase in plant defense. GTPase functions as molecular switch downstream of immune receptors, triggering immune responses and therefore leads to enhanced disease resistance^27^. Based on expressed sequence tags, 13 discrete unigenes encoding G-proteins including small GTPases were previously detected in *C. truncatum* (reclassified to *C. lentis* Damm, *sp. nov.*)—infected *L. culinaris* leaves^13^.

Highly expressed genes were also identified in the susceptible RIL LR-66-524 and clustered primarily in Clusters 1 (associated with cell-death functions), 2 (enriched for genes involved in water deprivation) and 4 (many genes related to cell growth and development processes). Cell death in the form of a hypersensitive response (HR) or programmed cell death (PCD) is a highly effective defense strategy of plants against biotrophic pathogens as it deprives the pathogen of essential viable host plant cells^28^. However, thriving in the presence of cell death, this mechanism can also be manipulated by necrotrophic pathogens to effectively increase susceptibility^29^. In hemibiotrophs, HR could be fatal during the biotrophic phase, but may be promoted by the pathogen during the transition to the necrotrophic phase. This was shown in *C. lentis* (previously *C. trunatum*) where constitutive overexpression of the effector *CtNUDIX* in isolates arrested their development in the biotrophic phase, during which they induced an HR, whereas in wild-type isolates *CtNUDIX* was only expressed towards the end of the biotrophic phase^30^.

Comparison of the 477 DEGs identified with the five QTL intervals significantly associated with resistance to *C. lentis* race 0 previously identified in a SNP-based linkage map of the *L. ervoides* RIL population LR-66^10^ revealed 22 genes that had high or low expression in the partially resistant RIL LR-66-528 at 24, 48, 72 and 96 hpi. Among them, two genes with high expression during the biotrophic as well as the necrotrophic phases had the most obvious involvement in host resistance. *Lc23518* was identified as an LRR receptor-like kinase, genes of which form one of the largest family of plant receptors, and its up-regulation at the necrotophic phase (96 hpi) in the partially resistant RIL was also validated by qPCR (Supplementary Table S4). The gene *Lc05911* contained a motif that encodes for a DEAD-box ATP-dependent RNA helicase. Overexpression of the DEAD-box ATP-dependent RNA helicase *OsBIRH1* in transgenic *Arabidopsis* showed enhanced disease resistance against *Alternaria brassicola* (necrotroph) and *Pseudomonas syringae* (biotroph)^31^. Overall, low numbers of overlapping genes between these two approaches may be a reflection of the different incubation times associated with QTL mapping (168 hpi) and the transcriptome study (24 to 96 hpi), but may also have been affected by using the *L. culinaris* genome for alignment.

Whether and to what degree these genes represent targets for resistance breeding remains to be determined. The ideal candidate would be a host receptor that interacts with a pathogen molecule during the biotrophic phase and arrests further development of *C. lentis* at this stage, or reduces the number of infections that successfully transition into the destructive necrotrophic phase. A candidate of interest for further explorations would be *Lc33118*, the cytokinin receptor histidine kinase exclusively expressed at 24 hpi in the partially resistant RIL LR-66-528, and its potential interaction with PR-1 gene *Lc37093*. Other targets would be the numerous LRR receptor-like kinase, in particular those detected early during the infection process. Further exploration of the filtered RNA-seq reads that did not align with the *L. culinaris* or will not align with the *C. lentis* genome, but may align to the *L. ervoides* genome could also reveal unique resistance gene candidates not present in the *L. culinaris* genome, considering that the latter appears to lack effective resistance against race 0 of *C. lentis*.

## Materials and methods

### Plant materials and inoculations

*Lens ervoides* RILs LR-66-524 with an average anthracnose severity of 72% and LR-66-528 with a severity of 5% were selected based on their reactions to inoculation with *C. lentis* isolate CT-30 in a previous study^10^. Four seedlings were grown in each 10 cm pot filled with a 3:1 mix of Sunshine #4 (Sun Gro^®^ Horticulture, Alberta, Canada) and perlite (Special Vermiculite Canada, Winnipeg, Canada) in a growth chamber (Conviron GR48, Controlled Environments Limited, Winnipeg, Manitoba, Canada) at 22 °C (day)/16 °C (night) with a 16 h photoperiod. Plants were fertilized with a complete fertilizer solution (PlantProd^®^ 20-20-20 plus micronutrients, Premier Tech Home &Garden Inc., Brantford, Ontario) 11 days after planting at a rate of 3 g L^−1^ H_2_O. The experiment was conducted in a randomized complete block design with three biological replications each represented by one pot with four seedlings. These were spray-inoculated with *C. lentis* isolate CT-30 (race 0) at a concentration of 5 × 10^4^ conidia mL^−1^. Due to space and sampling time constraints, replicates were blocked over time. Sampling was conducted on mock (water)-inoculated plants and on inoculated plants 6, 12, 24, 48, 72, 96, 120 and 144 h post-inoculation (hpi) and leaflets were fixed and stored in ethanol (histopathology) or stored in − 80 °C (RNA-seq).

### Histopathological study

Sampled leaves were fixed and stored in CMAA fixative (30% chloroform, 60% methanol, 10% acetic acid) at room temperature. Inoculated leaflets were treated with CMAA fixative twice or thrice until the leaflets were cleared before storage in 95% ethanol at room temperature. Six arbitrary leaflets rehydrated through decreasing ethanol concentrations of 70% (1 h), 50% (1.5 h) and 30% (1.5 h), stained with 0.05% Trypan blue and stored in 50% glycerol. Leaflets were mounted and were examined visually under a Zeiss Axioskop 40 microscope (Zeiss, Oberkochen, Germany) at 400X magnification. Pictures were taken using a Pixelink A686C camera (Pixelink A Navitor Company, Ottawa, ON, Canada) and Zeiss Axiovision software (Version 4.8.2 plus measurement module). The number of infection vesicle (IV)/primary hyphae (PH) formed per 25 appressoria, the percentage of leaflet area covered by acervuli per leaflet and the percentage area of dead tissue per leaflet were visually estimated.

Data were analyzed using SAS statistical package (Version 9.4, SAS Institute, Cary, NC, USA). Homogeneity of variances were assessed with the Levene’s test and normaility of error distribution with the Shapiro test. Data was analyzed with a repeated measures model where RILs and time points were considered fixed effects, time points were identified as repeated measure, and replications were considered random effects. At different time points, RILs were compared by multiple comparisons of means using Fisher’s least significant difference.

### Assessment of fungal biomass

For *in planta* fungal biomass determination, genomic DNA was extracted from 100 mg finely ground samples of inoculated and non-inoculated plants at 6, 12, 24, 48, 72, 96, 120 and 144 hpi using Qiagen DNeasy Plant Mini Kit^®^. The purity and quantity of DNA was determined using a NanoDrop ND8000 (Thermo Fisher Scientific, Wilmington, USA) (A260/280 < 2.0). For qPCR, previously designed primers were used to amplify the housekeeping gene Elongation factor 1-alpha of *L. culinaris* (*LcEF1-α*: forward TGTCGACTCTGGGAAGTCAA, reverse CTCTTTCCCTTTCAGCCTTG)^13^ and ACTIN of *C. lentis* (*ClACT*: forward CACGCTCTACTACGACGGAC, reverse GAAGACGAAGTTGTCGGGA)^32^. Primers were validated for their specificity using genomic DNA of IG 72,815, L01-827A, LR-66-528 and LR-66-524 in a C1000TM Thermal Cycler (Bio-rad Laboratories, Inc., Hercules, CA, USA) and by testing amplification efficiency using qPCR in a QuantStudio 3 System (Applied Biosystems Inc., Foster City, CA, USA) for a total of 5 dilutions for three biological replicates of mock samples of both RILs. Genomic DNA was adjusted to 25 ng µL^−1^. Each qPCR reaction contained 2 µL DNA template, 5 µL SYBR® Green (Catalog no. 4309155, Thermo Scientific), 0.2 µL of each 10 µM forward and reverse primers, and 2.6 µL sterilized water. The qPCR amplifications were performed in a QuantStudio 3 System using a fast-run program with default settings. The qPCR data of *ClActin* were normalized using *LcEF1-α* as a reference gene and log2 fold change (2^−ΔCT^) were used for statistical analyses following the method of Livak & Schmittgen^33^. The Student’s *t* test was used to determine the differences among the genotypes at different time-points (p-value < 0.05).

### Identification of differentially expressed genes

Total RNA was extracted and purified from three biological replicates per time-point (24, 48, 72 and 96 hpi) of CT-30-inoculated plants of LR-66-528 and LR-66-524, and from three biological replicates of non-inoculated plants using the Qiagen RNeasy Plant Mini Kit (with on-column DNAse treatment). The RNA integrity was determined by denaturing agarose gel electrophoresis and its quality and quantity were determined using NanoDrop ND8000 and Agilent 2100 Bioanalyzer (Agilent Technologies, Santa Clara, CA, USA). Strand-specific RNA libraries (adapted from the NEB Ultra-Directional RNA Library Prep protocol) were generated and sequenced using the Illumina PE125 HiSeq 2500 (v4 chemistry) at the Michael Smith Genome Sciences Center (BCGSC), BC, Canada. Raw fastq reads were filtered in Trimmomatic (version 0.36)^34^ to remove Illumina adaptors (TruSeq3-PE-2.fa:2:30:10) and leading and trailing low quality or N bases (below quality 3) (leading:3 and trailing:3), to cut when the average quality per base dropped below 15 after scanning of read with a 4-base wide sliding window (slidingwindow:4:15) and to drop reads of less than 36 bases (minlen:36). Removal of low-quality reads and adaptors was confirmed using FASTQC. Cleaned reads were submitted to Spliced Transcripts Alignment to a Reference (STAR version 2.6.1a; default settings)^35^ for mapping against the pre-release version of the *L. culinaris* genome version 1.2^36^. The read counts per gene were determined using STAR during the mapping process.

Variability of gene expression among the sequencing samples was estimated through Principal Component Analysis (PCA) by feeding gene read counts as the input file into JMP genomics 8.0. The proportion of variability accounted by RILs, incubation time, their interaction and replication was estimated through Principal Component Variance Analysis (PVCA) in the same program.

Comparisons were then made between non-inoculated mock and inoculated sample data from 24, 48, 72 and 96 hpi for each RIL to identify genes that responded to CT-30 infection using the R package DESeq2 at Padj < 0.05 and gene expression fold change > 2 with the raw counts file for all genes obtained using the STAR aligner as an input file. Pair-wise comparisons between LR-66-528 and LR-66-524 at 24, 48, 72 and 96 hpi were conducted on disease-responsive genes to identify DEGs between the two RILs for each time-point, using a threshold P_adj_ < 0.05 and gene expression fold change > 2, and were visualized in a Venn diagram generated with jvenn^37^.

### Comparison of transcriptome and QTL mapping data

Previously identified QTLs (2-LOD) intervals associated with anthracnose resistance^10^ were projected onto the physical map of *L. culinaris*^36^ using the software Strudel (v 1.15.08.25)^38^. The main region on the physical map with high density projections was focused, whereas minor regions projecting to other chromosome were not taken into consideration in order to restrict the distance. Using the GTF file of the *L. culinaris* genome, the markers spanning the highly dense region were used to identify the genes present in those intervals. These were compared with DEGs identified by RNA-seq.

### Gene expression profile analysis

Read counts per gene data procured using the STAR aligner were normalized by transforming to Reads Per Kilobase of exon per Million reads (RPKM). The genes with read counts less than 10 were removed before normalization. The resulting file was submitted to JMP genomics 8.0 for K-mean clustering analysis and GO enrichment analysis of genes in each of the resulting clusters. The *L. culinaris* gene IDs were transformed to the *M. truncatula* gene IDs because the *L. culinaris* was not completely annotated. The Entrez IDs of the corresponding genes were used as the input file for the gene enrichment analysis specifying *Medicago truncatula* as the input species (http://plantregmap.cbi.pku.edu.cn/go.php). The biological process aspect was selected for analysis at threshold False Discovery Rate (FDR) value (< 0.001) calculated with the method developed by Benjamini and Hochberg^39^.

### Validation of gene expression using quantitative PCR

Reproducibility of results was assessed by validating the expression of 21 selected DEGs (based on their potential role in disease resistance) at 24, 48, 72 and/or 96 hpi (depending on significant fold changes in the RNA-seq analysis, Supplementary Table S4) through RT-qPCR. Samples of LR-66-528 and LR-66-524 from an additional, independent experiment conducted as described above were generated, and total RNA was extracted from samples collected at 24, 48, 72 and 96 hpi, and non-inoculated RILs as described earlier. RNA of all the samples were adjusted to 50 ng L^−1^ to synthesize cDNA using the High-Capacity cDNA Reverse Transcription kit (Thermo Fischer Scientific, Wilmington, USA). Elongation factor 1-alpha was used as the reference gene. Primers were designed with Primer3 (web version 4.1.0) using default settings from the exonic regions of the selected genes based on the lentil genome. Amplification efficiencies were tested as described above using cDNA (50 ng/μl) serially diluted 1:2 (V/V) 4 times in nuclease-free ultra pure water (Invitrogen Life Technologies, Carlsbad, CA, USA) and were calculated based on the slope of the standard curve with equation E = − 1 + 10 (− 1/slope). Each RT-qPCR reaction consisted of 2 μL cDNA template, 5 μL SYBR green, 0.8 μL of each forward and reverse primers and 1.4 μL of nuclease-free water. RT-qPCR was performed in QuantStudio 3 System using a fast-run program with default settings. Fold changes in gene expression in LR-66-528 and LR-66-524 compared to that in mock-inoculated samples were calculated as described by Schmittgen and Livak using *EF1-α* as the reference gene^40^. Student *t* tests were used to determine significant differences at *P* < 0.05 in gene expression (2^−ΔCT^) between partially resistant and susceptible RILs after confirmation of significant differences with respective mock-inoculated samples.

### Guideline statement

All experimental research complies with relevant institutional, national, and international guidelines and legislation.

## Supplementary Information

Supplementary Tables.

## Data Availability

RNA-seq data of this study can be found in the NCBI BioProject Repository accession id: PRJNA784980.
